# The Relationship of Sleep with Temperature and Metabolic Rate in a Hibernating Primate

**DOI:** 10.1371/journal.pone.0069914

**Published:** 2013-09-04

**Authors:** Andrew D. Krystal, Bobby Schopler, Susanne Kobbe, Cathy Williams, Hajanirina Rakatondrainibe, Anne D. Yoder, Peter Klopfer

**Affiliations:** 1 Department of Psychiatry and Behavioral Sciences, Duke University School of Medicine, Durham, North Carolina, United States of America; 2 Lemur Center, Duke University, Durham, North Carolina, United States of America; 3 Department of Animal Ecology and Conservation, Hamburg University, Hamburg, Germany; 4 Lemurs Biomedical Monitoring Environmental Department, Biodiversity Section, Ambatovy, Mineral Society Anonym, Antananarivo, Madagascar; 5 Departments of Biology and Evolutionary Anthropology, Duke University, Durham, North Carolina, United States of America; 6 Department of Biology, Duke University, Durham, North Carolina, United States of America; University of Sydney, Australia

## Abstract

**Study Objectives:**

It has long been suspected that sleep is important for regulating body temperature and metabolic-rate. Hibernation, a state of acute hypothermia and reduced metabolic-rate, offers a promising system for investigating those relationships. Prior studies in hibernating ground squirrels report that, although sleep occurs during hibernation, it manifests only as non-REM sleep, and only at relatively high temperatures. In our study, we report data on sleep during hibernation in a lemuriform primate, Cheirogaleus medius. As the only primate known to experience prolonged periods of hibernation and as an inhabitant of more temperate climates than ground squirrels, this animal serves as an alternative model for exploring sleep temperature/metabolism relationships that may be uniquely relevant to understanding human physiology.

**Measurements and Results:**

We find that during hibernation, non-REM sleep is absent in Cheirogaleus. Rather, periods of REM sleep occur during periods of relatively high ambient temperature, a pattern opposite of that observed in ground squirrels. Like ground squirrels, however, EEG is marked by ultra-low voltage activity at relatively low metabolic-rates.

**Conclusions:**

These findings confirm a sleep-temperature/metabolism link, though they also suggest that the relationship of sleep stage with temperature/metabolism is flexible and may differ across species or mammalian orders. The absence of non-REM sleep suggests that during hibernation in Cheirogaleus, like in the ground squirrel, the otherwise universal non-REM sleep homeostatic response is greatly curtailed or absent. Lastly, ultra-low voltage EEG appears to be a cross-species marker for extremely low metabolic-rate, and, as such, may be an attractive target for research on hibernation induction.

## Introduction

The specific functions of sleep remain unknown. It has been hypothesized that sleep may play a role in the regulation of temperature and metabolism based on several lines of research suggesting that these phenomena are highly inter-related. This includes studies of prolonged deprivation of REM and non-REM sleep and total sleep deprivation in the rat, [1]–[2] a study of 60 hours of total sleep deprivation in humans where a decrease in core body temperature was found, [3] single night sleep deprivation studies demonstrating increases in appetite and release of ghrelin, a hormone that stimulates hunger, [4] and studies of chronic partial sleep loss and intermittent total sleep deprivation in rats and humans where these interventions led to changes in food intake, weight, glucose tolerance, insulin sensitivity, energy expenditure, ghrelin levels, and leptin levels (a hormone that suppresses appetite). [5]–[8] An additional link of sleep and temperature regulation is that many mammals suspend their usual regulation of temperature during REM, though in humans it is only decreased compared with other states. [9] There is also evidence that non-REM sleep has important ties to cellular metabolic activity. Adenosine is a molecular by-product of metabolic activity, produced when energy stored in the form of adenosine triphosphate (ATP) is consumed. [10]–[11] When adenosine has been injected into the basal forebrain region of several species of mammals it triggers non-REM sleep. [10]–[13] Evidence that this mechanism plays a functional role in mediating sleep is that adenosine levels in the basal forebrain build up in proportion to the degree of prior waking and decrease over the course of a night of sleep. [13] Further, in fruit flies and mammals, waking and short-term sleep deprivation up-regulate metabolism-related genes [14]–[15].

Based on such observations, it has been hypothesized that the regulation of temperature and metabolism are intimately related to the ultimate functions of sleep and that the study of sleep during hibernation, a state where metabolic rate dramatically decreases and core body temperature drifts towards ambient, is of particular interest for elucidating these relationships. [16]–[17] However, few studies of sleep in hibernating animals have been carried out and the majority of this work has involved ground squirrels.

In ground squirrels REM sleep appears to be absent during hibernation and non-REM sleep is more likely at higher temperatures, such that continuous non-REM sleep is seen during hibernation at moderate temperature. [18]–[19] Periodic arousals to euthermia are also reported in ground squirrels hibernating at low temperature where sleep does not occur and some have hypothesized that this occurs to allow sleep which is a necessity. [20] EEG slow-wave activity recorded during sleep during arousals occurs in a pattern that is consistent with the hypothesis that ground squirrels are sleep deprived when they emerge from torpor bouts in proportion to the length of the torpor bout and in inverse proportion to the time slept during hibernation. [18]–[23] However, several studies which prevented sleep from occurring during arousals from torpor bouts demonstrate that this EEG slow-wave activity is not, in fact, a manifestation of sleep deprivation following periods of torpor. [24]–[25] Thus, the purpose of the period arousals remains unknown and there is evidence that torpor eliminates or decreases the usual need for sleep as manifested in the homeostatic build-up of sleep drive that develops when sleep does not occur over time.

Thus, the studies carried out in hibernating animals are consistent with research in euthermic animals which suggest that temperature, metabolic rate, and sleep are highly inter-related. This work further suggests, however, that these relationships may be sleep stage specific: REM does not seem to occur when homeothermy is suspended and the likelihood of non-REM sleep seems to be correlated with temperature and metabolic rate. There is also evidence that the decrease in body temperature and metabolic rate that occurs during hibernation is accompanied by a decrease in the usual homeostatic drive for sleep.

In order to further study the relationship between temperature regulation, metabolism, and sleep, we obtained the first EEG records in both the non-torpid sleeping state, and during hibernation, in fat-tailed dwarf lemurs (Cheirogaleus medius). These animals, who have only recently been documented to undergo hibernation, hold promise for providing information on the sleep-temperature/metabolism relationship that is uniquely relevant to humans as the dwarf lemurs and their close relatives are the only primates and closest genetic relatives to humans known to hibernate for prolonged periods. [26]–[27] Further, studying these animals is particularly likely to provide insights into the sleep-temperature/metabolism relationship as they experience substantial daily variation in ambient temperature and metabolic rate during hibernation. [26]–[27] As the dwarf lemurs are tropical hibernators, the study of these animals also allows examination of sleep during hibernation at relatively higher ambient temperatures than prior work. Based on the differences of these animals from previously studied hibernators, we sought to test the hypothesis that the relationship of sleep with temperature and metabolic rate during hibernation in these primate hibernators might differ from the non-primate hibernators studied to date.

## Materials and Methods

### 1. Summary of Methods

This study was carried out in strict accordance with the recommendations in the Guide for the Care and Use of Laboratory Animals of the National Institutes of Health. The protocol was approved by the Institutional Animal Care and Use Committee of Duke University. All efforts were made to minimize animal suffering and no animals were sacrificed in the conduct of this research. Methods were limited by the fact that we worked with an endangered primate. Because of this status we were precluded from performing many invasive or manipulative procedures that would have yielded useful information. In addition, the animals we studied are found in an exceedingly difficult environment, physically and politically, and there exists but a single, small breeding colony (less than a dozen adults) in captivity. However, because Cheirogaleus is the sole primate known to undergo long-term hibernation, we believe these data, though limited, are an invaluable addition to our store of information about hibernation.

Scalp electroencephalographic (EEG) recordings were obtained on 10 Cheirogaleus medius (C. medius) in 4 conditions: 3 during sleep in non-hibernating season; 5 during hibernation season in the wild; 1 during hibernation in the wild with simultaneous metabolic rate measurement; 1 during hibernation in the Duke University Lemur Center (DLC) in North Carolina, USA at nearly constant temperature with simultaneous metabolic rate measurement.

The non-torpid sleep data were obtained from diurnally active animals at the DLC. The DLC is ALAAC certified and has been especially commended for its animal care. The animals studied were housed in free-range rooms that simulate their natural environment. They were provided a diet which varied and consisted largely of natural fresh fruits and vegetables. The data obtained from animals hibernating in the wild were recorded in Kirindy Forest, a nature preserve on the west coast of Madagascar, some 60 km north of the town of Morondava at latitude 20 south. A permit for carrying out this work in Kirindy Forest was obtained from the Ministry of Agriculture of Madagascar. The forest is a very dense, second growth, dry deciduous forest, with an average crown height of 10–15 meters. The area has a pronounced seasonality, the summers being hot and wet, winters cold and dry. Winter days may have a temperature range of 5 to 30 degrees C. Both the vegetation on which the lemurs depend and water holes are in very short supply during most of the winter. The fat-tailed lemurs ordinarily nest and hibernate in tree hollows, usually at some height from the ground, but can be induced to use nest-boxes fastened to trees, which was the case with most of the animals we studied. These nest boxes varied from 15×15×15 cm to 20×15×15 cm.

### 2. EEG Recording Methods

For EEG recording, the animal was removed from its cage, nest, or nestbox, anesthetized using ketamine 10mg/kg (Ketaject, 100 mg/ml Bioniche Teoranta, Inverin, Co. Galway, Ireland) and midazolam 0.25 mg/kg (Midazolam 5 mg/ml, Bedford Laboratories, Bedford, Ohio USA). For both recordings carried out with metabolic measurement and one of the recordings carried out in the DLC during sleep in non-hibernation season, EEG data were obtained via four subdermal needle electrodes (Grass-Telefactor, West Warwick, RI USA) inserted separately into each quadrant of the cranium: right, left, rostral and caudal. For the remainder of the animals, the scalp was shaved and Ag/AgCl scalp EEG electrodes were placed with electrode paste (Grass-Telefactor, West Warwick, RI, USA). Leads were held in place using an adhesive semipermiable membrane (Tegaderm, 3 M Health Care, St. Paul, MN, USA) which was then wrapped in flexible cohesive bandage (Webtear, Webster Veterinary) and secured with bandage tape (Wet-Pruf ® The Kendall Co., Mansfield, MA, USA). The leads were left in place for up to 6 days as long as technically acceptable recordings were obtained. For all animals electro-oculographic (EGO) and electromyographic (EMG) data were obtained in addition to the EEG data.

EOG data were obtained from two leads one with Grid 1 of the differential amplifier recording from the left anterior region of the scalp and the other with Grid 1 in the right anterior region and Grid 2 for both leads placed in the posterior of the head. As a result, these leads sensitively picked up eye-movement related electrical potentials which are largest in the front of the head. EMG activity was recorded from scalp muscles and sampled with all of our electrodes. For the needle electrodes the needles were embedded directly through the scalp muscles with the tips underneath the muscles and the shaft of the needles maintaining direct contact with scalp muscle tissue. The scalp electrodes resided just over scalp musculature. The anterior and posterior reference leads were far enough apart that cancellation of these potentials could not occur. We could easily note the presence of evident EMG in our waking animals which confirmed that we were able to reliably record EMG data. The signals were recorded using Compumedics Siesta (Compumedics Inc, Victoria, Australia) devices which included amplification and digitization at 256 Hz with 16 bit accuracy. Signals were recorded using a 250 mcv peak-to-peak scale. These devices wirelessly transmitted the data to a laptop computer for real time viewing and the data were saved on memory cards in the Siesta devices for off-line review and analysis. The data were reviewed and converted to European Data Format (EDF) using Compumedics EEG scoring and analysis software. The EDF files subsequently underwent further scoring and spectral analysis using software written by ADK which has been validated against MATLAB (MathWorks, Natick, MA, USA) software [28]–[29].

### 3. Measurement of Metabolic Rate

The rate of metabolism was measured as VO2 (rate of oxygen consumption) using a portable oxygen analyzer (OxBox, designed and constructed by T. Ruf & T. Paumann, FIWI, University of Veterinary Medicine Vienna) with chemo-electric sensors (Bieler & Lang, Achern, Germany; accuracy <0.02 vol. %). Oxygen sensors were calibrated directly before the field season in the laboratory using calibration gas made by a gas-mixing pump (Wölsthoff, Bochum, Germany, type G27). For the animal studied in the wild, the wooden nest-box, which was 20×15×15 cm and fixed to a tree, served as the metabolic chamber. For the animal studied in the DLC a hollowed out log approximately 2 feet in length and 5 inches in diameter served as the nest-box and metabolic chamber. Air Flow was measured at about 60 l/h. Flow rate was adjusted in relation to nest box size and so as to maintain <1% oxygen depletion between incurrent and excurrent air. Flow rates of comparable studies vary between 50 l/h and 70 to 90 l/h depending on the size of the nestbox or treehole. [26] To ensure that all consumed air was captured from the animal, we took the following precautions: 1. The small hole for the tube was placed opposite the entrance hole and the end of the tube was located next to the animal’s head (on the floor of the box). Thus, all air that was pumped through the chamber had to pass the animal before it was pulled into the tube. 2. Any crevices in the nestbox (except for a little air inlet) were sealed with tape to avoid that expired air could diffuse outside). The flow rate was continuously monitored by the mass flow meter of the gas analyser system. Sampled air was dried and filtered with silica gel before entering the gas analyzer. Oxygen content of the sample air was measured once per minute. To control for any drift of the oxygen sensor, reference air (surrounding air) was analyzed once per hour for 5 min (zero check). Metabolic rate was calculated as milliliters O2 per hour, correcting values for ambient pressure and Ta. For comparison with the metabolic rate presented here, we estimate that the mean basal metabolic rate of resting lemurs that are not in torpor in the field is 133 ml O2/h. This is based on the mean of data collected from 14 animals in post-absorptive resting lemurs while awake [26].

Nest-box temperature (T_n_) was recorded with a data logger (Thermochron IButton/DS1921G, Semiconductor, Dallas, USA) placed inside the nest-box. In the wild, T_a_ was also measured outside the nest-box by a temperature sensor, that was part of the gas analyser system. (placed at the shady side of a tree). We were unable to continuously monitor body temperature, but several previous studies had confirmed that C. medius animals in torpor (in nest-boxes, and unresponsive for days on end) tracked the environmental temperature to within ½ degree C [26].

This methodology was employed for 1 C. medius studied in the wild in Madagascar and one studied in the DLC. For the other 5 animals studied in the wild in Madagascar, metabolic measurement was not available and behavioral observation, occurring every 4 hours, was employed to determine whether signs of torpor were present (quiescence with eyes closed during the usual wake period, diminished respiratory rate [<20/min]). In addition several of these animals were handled during the recordings to replace electrodes etc. and non-responsiveness to handling and/or skin that was cold to the touch were noted.

### 4. Scoring of EEG Data

All EEG records were scored and analyzed by ADK who is Board Certified in Sleep Medicine (American Board of Sleep Medicine, American Board of Psychiatry and Neurology) and EEG (American Board of Clinical Neurophysiology, American Board of Psychiatry and Neurology). After having observed the presence of the classical features of mammalian sleep in the non-torpid C. medius studied in the Duke Lemur Center these features were used to stage all of the recorded data in 30 second epochs using standard scoring criteria with the exception that REM was operationalized as 30 second epochs where there was at least 1 rapid lateral eye-movements, there were no other movements, and Beta and Gamma EEG Spectral Power were below their median across all artifact-free epochs in the recording (as a proxy for decreased electromyographic activity; a hallmark of REM sleep). [30]–[31] The rapid eye movements of REM were detected based on several features including their characteristic polarity in recordings with the configuration of EEG leads that we employed. Because the eye ball is an electrical dipole which is cornea positive and retina negative, lateral eye movements result in electrical potentials which have an unmistakable appearance in our left and right anterior-to-posterior leads consisting of a phase reversal (identical or similar waveforms of opposite polarity). This allows unequivocal differentiation of lateral eye movement related potentials from brain generated potentials which are in phase in these leads. [30]–[31] We also used the morphology of the waveforms to identify them as rapid lateral eye movements as recommended by published scoring guidelines: bilaterally synchronous, irregular, sharply peaked waveforms with an initial deflection lasting less than 500 msec. [30]–[31] Lastly, we used distribution as they are picked up in anterior but not in our posterior leads. REM activity was distinguished from activity during quiet waking based on the standard criteria for scoring REM which requires a decrease in EMG amplitude, presence of Rapid Eye Movements, absence of EEG slow-waves, k-complexes, or sleep spindles, and the presence of characteristic EEG frequency content. [30]–[31] Waking was scored based on taking into consideration all available data including EEG frequency content, eye movements, gross body movements, and EMG. The critical feature distinguishing REM from quiet wake in this case was the decreased EMG amplitude [30]–[31].

We also measured and present data on the number of eye movements for each 30 second epoch that was scored as a REM sleep-like state to provide further information to the reader about the detailed characteristics of these periods given the novelty of our observation of such activity occurring during periods of torpor.

## Results

### 1. EEG Data Recorded in Non-Torpid Sleep in C. medius

**Figure 1:**
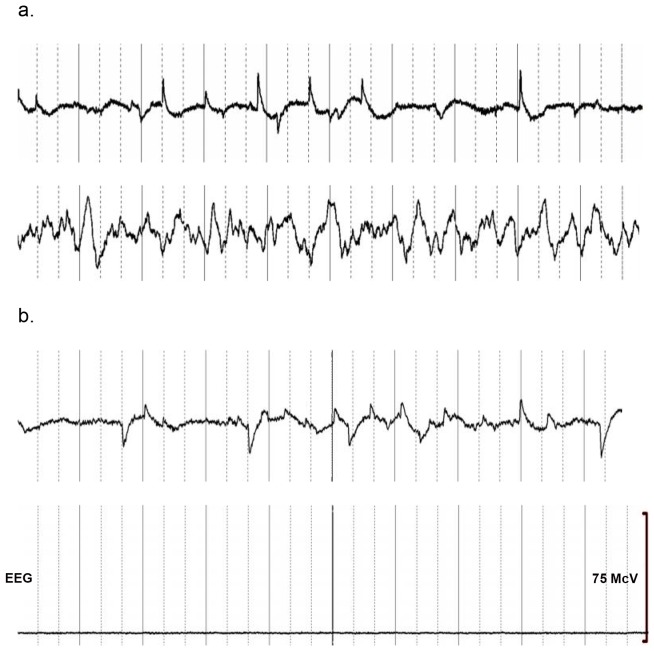
Four 30 second segments of EEG Data. A. Two segments of data recorded in non-torpid sleep in Duke Lemur Center. The top segment displays EEG data during a period of REM sleep. The display scale is 250 µV from the top to the bottom. The bottom segment displays EEG data during a period of Non-REM sleep. The display scale is 150 µV from the top to the bottom. b. Two 30 second segments of EEG data recorded during torpor in the wild verified by decreased metabolic rate. The top segment includes REMs. The display scale is 250 µV from the top to the bottom. The bottom segment is typical monotonous very low-voltage activity seen through much of these recordings and the display scale is 75 µV from the top to the bottom.

The EEG data manifested the classical features of mammalian sleep comprised of non-rapid eye movement (NREM) sleep associated with an increase in low-frequency EEG activity and REM sleep marked by decreased EMG activity and rapid eye-movements (See Figure 1).

### 2. EEG Data Recorded During Torpor Documented with Metabolic Rate Measurement

**Figure 2:**
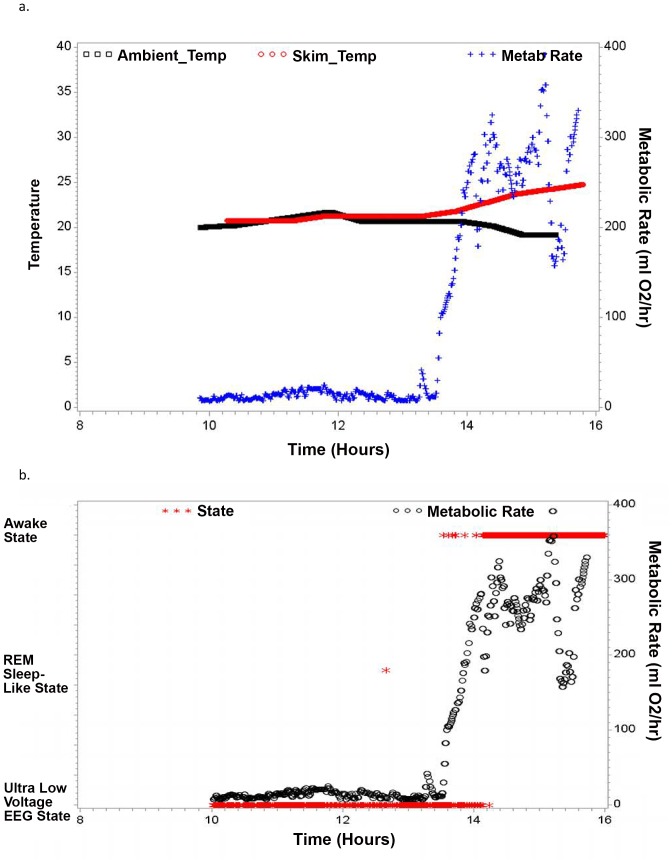
Data from recordings carried out for a torpid C. medius studied in the Duke Lemur Center. Simultaneous EEG, Temperature and Metabolic Rate recordings were obtained. a. Ambient Temperature, Skin Temperature, and Metabolic Rate vs Time of Day in Hours. Arousal from torpor is evident at approximately 13.5 hours. b. State (derived from visual scoring of the data in 30 second epochs) and Metabolic Rate vs. Time of Day in Hours.

We studied one C. medius during hibernation in the DLC where we documented torpor in terms of diminished metabolic rate during 7 hours of recording at essentially constant environmental temperature during which metabolic rate was dramatically decreased compared with non-hibernation metabolic rate and temperature tracked the ambient temperature (see Figure 2). The maximum metabolic rate noted during the period of torpor was 30 (ml O2/hr), substantially lower than the non-torpid state where metabolic rate was >150 ml O2/hr and typical of torpor in C. medius. [26] After approximately 5 hours of recording, metabolic rate increased, body surface temperature became greater than the ambient temperature, and behavior consistent with arousal from torpor was noted (See Figure 2). We were able to record continuous EEG data in this animal during the 5 hours of torpor and 2 hours following arousal from torpor. The EEG data were marked by long periods of very low voltage (<5 mcV) monotonous EEG activity interspersed with occasional spindle activity during torpor. There were also a few brief periods of REMs associated with very low amplitude EMG activity and absence of movement (see Figure 2). The EEG signal amplitude was decreased compared to the EEG obtained during and after arousal from torpor and was significantly correlated with metabolic rate (p<0.0001) (see Table 1). The EEG data recorded during periods marked by REMs during torpor appear to be the same as what we recorded during REM sleep during the non-hibernating season in the DLC in C. medius (See Figure 1). There was no evidence for signs of non-REM sleep at any point during the recording as seen in the recordings carried out during sleep in the DLC (see Figure 1).

**Table 1:** Statistical Analysis of Relationships of EEG Activity to REMs, Metabolic Rate, and Temperature in 7 Animals Recorded During Torpor.

Lemur	Site	Metabolic Recording	Recording Duration	Correlation of Ambient Temperature and Total EEG Power	Correlation of Metabolic Rate and Total EEG Power	Correlation of Ambient Temperature and Number of Rapid Eye Movements per 30 second epoch	Mean Ambient Temp REM Epochs (°C)	Mean Ambient Temp Other Epochs (°C)	Beta Power REM Epochs (µV^2^)	Beta Power Other Epochs (µV^2^)	Gamma Power REM Epochs (µV^2^)	Gamma Power Other Epochs (µV^2^)
**1**	In Wild	Yes	5 Days	0.23 (p<0.0001)	0.30 (p<0.0001)	0.35 (p<0.0001)	27.26 (4.9)	19.75*** (6.5)	0.34 (0.057)	0.73*** (0.73)	0.0040 (0.007)	0.042*** (0.063)
**2**	In Wild	No	2 Days	0.30 (p<0.0001)	N/A	0.32 (p<0.017)	30.00 (6.4)	18.48*** (7.89)	0.053 (0.002)	0.062* (0.34)	0.00068 (0.00015)	0.0032***(0.0037)
**3**	In Wild	No	1 Day	0.21 (p<0.0001)	N/A	0.23 (p<0.07)	26.40 (4.97)	20.49*** (8.81)	0.074 (0.032)	0.16 (0.59)	0.00084 (0.00019)	0.0012* (0.0012)
**4**	In Wild	No	3 Days	−0.13 (N.S.)	N/A	0.21 (p<0.02)	24.46 (7.22)	20.46*** (7.08)	0.13 (0.032)	0.24*** (0.16)	0.0050 (0.0017)	0.0081***(0.0087)
**5**	In Wild	No	2 Days	0.015 (N.S.)	N/A	0.25 (p<0.04)	31.48 (1.15)	19.61*** (7.92)	0.27 (0.096)	0.48*** (0.54)	0.0030 (0.00085)	0.0063 (0.346)
**6**	In Wild	No	2 Days	−0.04 (N.S.)	N/A	0.28 (p = 0.079)	30.02 (3.89)	27.2* (6.72)	0.3 (0.059)	1.04* (1.98)	0.0047 (0.0012)	0.033* (0.071)
**7**	DLC	Yes	6 Hours	−0.12 (N.S.)	0.66 (p<0.0001)	N/A	21.2 (0.71)	20.5 (0.64)‡	0.20 (.0049)	1.32 (1.81)‡	0.0019 (0.0033)	0.032 (0.092) ‡

* p<0.05;

** p<0.01;

*** p<0.0001 For Difference between REM Epochs and Epochs Other Than REM; DLC = Duke Lemur Center;

‡ Only 2 REM epochs were included in these analyses.

**Figure 3:**
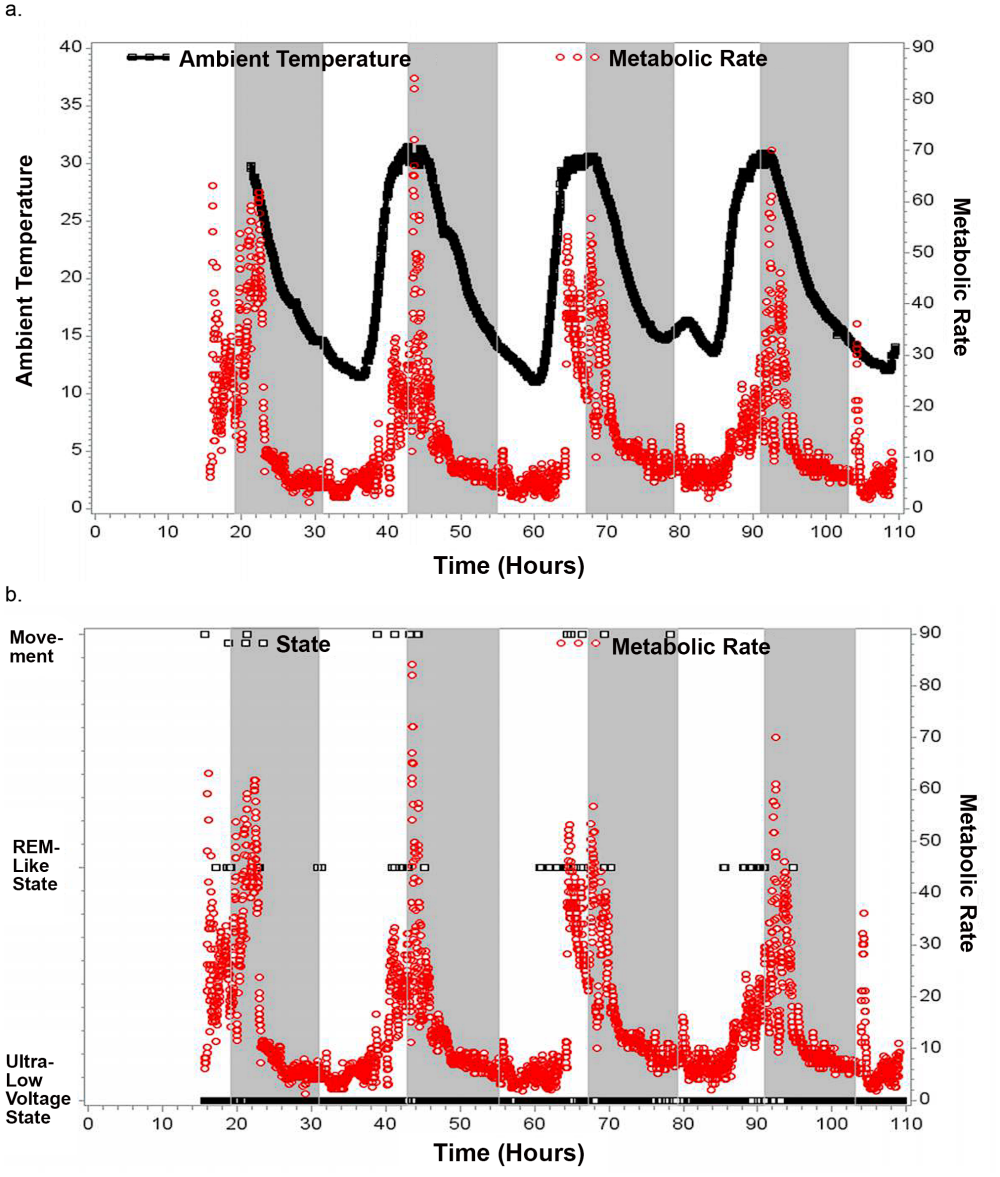
Data collected in a C. medius Studied in a Nest Box in the Wild in Madagascar With Continuous Torpor Verified by Metabolic Rate. The gray shading indicates periods of darkness. a. Ambient Temperature and Metabolic Rate vs Time of Day in Hours. b. State scored for each 30 seconds during the period of monitoring (it was either a monotonous ultra-low voltage state, a REM-like state, or brief periods of movement which obscured making a state determination designated as “movement” epochs) vs Metabolic Rate vs Time of Day in Hours. c. Rapid eye movements (REMs) per 30 second epoch and Metabolic Rate vs. time in hours. c. Beta power (16.5–30 Hz) per 30 second epoch and Metabolic Rate vs. time in hours d. Delta power (0.5–3.5 Hz) per 30 second epoch and Metabolic Rate vs. time in hours. For reference, the mean basal metabolic rate of resting lemurs that are not in torpor in the field is 133 ml O2/h. This is based on the mean of data collected from 14 post-absorptive resting lemurs while awake. The lowest basal metabolic rates we measured in non-hibernating lemurs were ≥115 ml O2/hr^26^.

**Figure 4:**
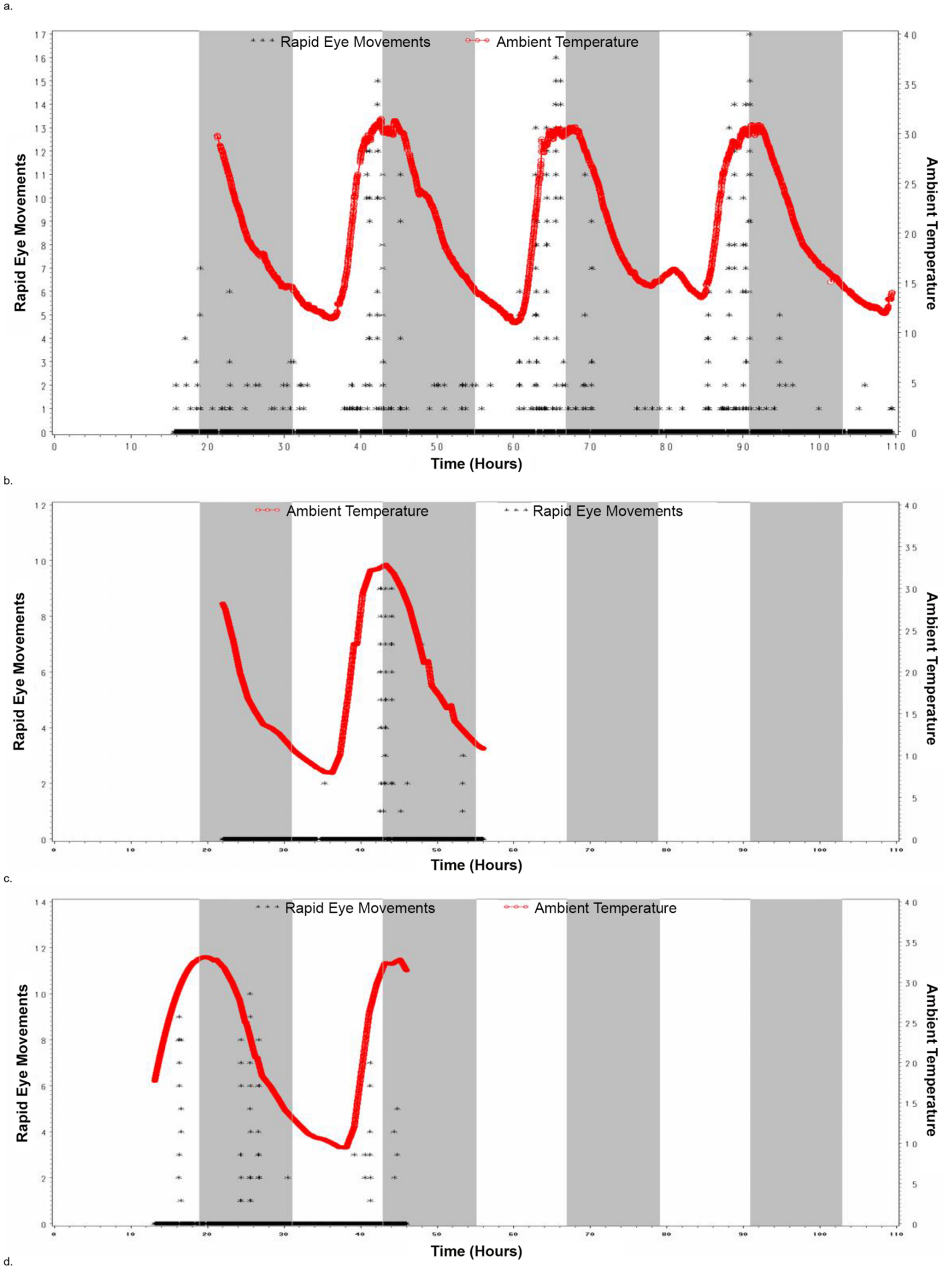
REMs per 30 second epoch and Ambient Temperature vs Time of Day in Hours in 6 C. medius studied in the wild in Madagascar. The top panel of the figure represents data from the animal documented to be in torpor with metabolic measurement. Note: the periods where EEG data were available for each animal coincides with the time-points where Ambient Temperature data is included in the graphs. Graphs a-f depict these data for Lemurs 1–6 respectively as designated in Table 1. Note: Because we have plotted a data point for every 30 second period over the roughly 4 days of data depicted, data from adjacent 30 second epochs which have the same or nearly the same value will appear to be on top of each other.

We also recorded data from one C. medius during hibernation in the wild for 5 days where we documented torpor in terms of diminished metabolic rate which varied proportionally to ambient temperature (see Figure 3). The maximum metabolic rate noted during this 5 day period was 90 (ml O2/hr), which is reduced compared with the non-torpid state and typical of torpor in C. medius. [26] We were able to record continuous EEG data in this animal during 5 days of torpor. The EEG data were marked by long periods of very low voltage (<5 mcV) monotonous EEG activity interspersed with occasional spindle activity. This pattern was particularly likely when temperature and metabolic rate were low (see figure 3) such that the total power in the EEG signal was significantly correlated with both temperature and metabolic rate (p<0.0001) (see Table 1 and Figure 3). We also observed periods of rapid lateral eye movements (REMS) associated with very low amplitude EMG activity and absence of movement. This activity was particularly likely when temperature was relatively high (mean temperature during epochs where REM sleep-like activity occurred was 27.3 deg C, whereas the mean for all other epochs was 19.8 deg C; p<0.0001) (See Table 1). It also was not restricted to the usual sleep period for these animals (see figures 3 and 4). Further, the number of rapid-eye movements per 30 second epoch (REM density) was significantly correlated with temperature (r = 0.35; p<0.0001) (See Figure 4 and Table 1). The period of increased temperature coincided with the last half of the usual sleep period and the early portion of the usual wake period. REMs also tended to occur prior to the daily peaks in metabolic rate such that 30 second epochs of REM occurred on average 2.09 hours (s.d. = 3.29) prior to the maximum metabolic rate each day whereas for all other 30 seconds of data the mean time that they occurred was 1.61 hours (s.d. = 7.75) after the maximum metabolic rate (p<0.0001). Higher frequency activity (beta and gamma power) was significantly lower during the REMs than during the periods of the recording excluding REMs (p<0.0001), though it should be noted that this reflects, at least in part, the manner in which REM epochs were defined (see Table 1; Methods). The EEG data recorded during REM periods during torpor appeared to be the same as what we recorded during REM sleep during the non-hibernation season in the Duke Lemur Center in C. medius (See Figure 1). Spectral analysis also revealed a relative increase in Delta power (although the amplitude of this activity was still quite low compared with Delta power seen in non-REM sleep in non-torpid lemurs) during 2 brief periods when metabolic activity was relatively low (See Figure 3). There was no evidence for signs of non-REM sleep at any point during the recording as seen in the recordings carried out in the Duke Lemur Center in non-hibernation season (see Figure 1).

### 3. EEG Data Recorded in Five C. medius Animals in Kirindy Forest Madagascar in the Wild without Metabolic Measurement

At least two signs of torpor (persistent quiescence during the usual wake period and decreased respiratory rate) were observed during EEG recordings in all of the 5 C. medius studied where metabolic measurement was not available. The EEG data obtained in these animals were essentially the same as what was observed in the animals where torpor was verified with metabolic measurement. The EEG was marked by long periods of monotonous very low-voltage activity interspersed with spindle activity which tended to occur at lower temperature, though the relationship between temperature and total EEG signal power was only significant in 2 of the 5 animals studied (See Table 1). The EEG was marked by intermittent periods of REMs occurring in the setting of an absence of movement and very low voltage or absent EMG activity and low signal amplitude in general (See Figure 2 and Table 1). These tended to occur with greatest frequency during periods of elevated temperature which coincided with the last half of the light period and beginning of the dark period (see Figure 4). Significantly greater ambient temperature was found during REM epochs vs all other epochs for all 5 of these animals (p<0.05) (See Figure 4 and Table 1). Rapid lateral eye movements occurring in association with closed eyes and behavioral quiescence were visualized twice during the usual C. medius wake period during behavioral observation which took place every 4 hours while EEG data were being recorded. There was also an association between the number of rapid-eye movements per 30 second epoch (REM density) and temperature which was significant for 3 of the 5 animals studied (p<0.05) and there was a trend for a significant relationship in the other two animals (p<0.10) (See Figure 4 and Table 1). Slow-wave sleep like activity was minimal in these 5 animals confirming what was seen in the animal undergoing metabolic measurement.

## Discussion

The principal findings of our study are that during hibernation in the primate C. medius non-REM sleep is essentially absent, even at higher ambient temperatures, and daily periods of REM sleep-like activity occur in conjunction with relatively high ambient temperature. In addition, at relatively low metabolic rate we observed monotonous very low-voltage EEG activity interspersed with occasional spindle activity. These findings were observed in one animal verified to be in torpor over 5 days and one verified to be in torpor for 5 hours with metabolic measurements as well as 5 other animals that manifested at least two signs of torpor where the same findings were evident, although metabolic confirmation of torpor was not possible.

These results are notable in that they appear to suggest that the findings in the primate C. medius are not consistent with what has been previously reported in non-primate hibernators. [18]–[25] In fact, from a sleep stage point of view, our findings appear to be the polar opposite of what has been reported in the ground squirrel, where REM sleep is absent during torpor and non-REM sleep is only seen at temperatures above 10 degrees C which is above the environmental temperature in which torpor occurs in most small hibernators. However, the apparent difference noted between C. medius and non-primate hibernators must not be taken to be definitive because of limitations in our work, some of which reflect that we were working with an endangered primate which precluded any invasive procedures. The most important limitation of our work is that we studied a relatively small number of animals and we lacked metabolic rate and body temperature measures in all but 2 of the lemurs studied. When such measures were absent we had no means independent of the EEG/EMG/EOG data to confirm the state of the animals (waking, sleep, or torpor).

It will be important to carry out studies with a larger number of animals where metabolic rate and temperature are monitored along with EEG data because of the potential implications of this work. One important conclusion suggested by a difference in the dwarf lemur and ground squirrel is that the relationship of sleep stage with temperature, metabolic rate, and the regulation of these phenomena may not be unique. Many studies have been carried out to establish the functions that REM and non-REM sleep may serve that might explain their widespread existence in the animal kingdom. [32]–[35] Such work may need to take into account the possibility that such functions and/or the factors which modulate these stages may vary across species or across mammalian orders. A fundamental difference between the dwarf lemurs and ground squirrels would also suggest that hibernation in primates is also not homologous to hibernation occurring in non-primates even though these states are phenotypically highly similar. If confirmed, the findings of our study would suggest that hibernation is probably not an ancient, conserved, trait but is a remarkable case of convergent evolution to an extreme physiological phenotype.

Several factors may explain why the findings of our study are so dramatically different than what has previously been observed with hibernating animals of other species. [18]–[25] This is the first time that sleep during hibernation has been studied in a primate and the physiology of sleep and regulation of temperature/metabolism in the primate may differ from that of the other mammals studied. Another important consideration is that C. medius is a relatively warm-weather hibernator that is exposed to significant daily variation in ambient temperature, unlike many of the other animals studied who are arctic hibernators, and this may affect a number of factors including the ongoing level of metabolism needed to maintain survival during hibernation. [10]–[13] Clearly, much additional work is needed to explain the different relationships between sleep and temperature/metabolism observed here and reported for non-primate hibernators.

At the same time, there were some important commonalities of our findings with what has been observed in prior studies of sleep in hibernation. These findings confirm that sleep is significantly inter-related with temperature and metabolic rate in that sleep is less likely the lower the ambient temperature and metabolic rate during hibernation. Further, as metabolic rate (and in 3 of the animals temperature) decreased, the EEG patterns we observed are the same as that noted in non-primate hibernators: monotonous ultra-low voltage EEG activity interspersed with occasional spindle activity. [19] Studies in other animals will be needed to determine the universality of these findings. However, we hypothesize that these findings reflect the general phenomenon that as temperature and metabolic rate decrease, neuronal activity must broadly diminish leading to significant decreases in fluctuations in membrane electrical potential and the resultant decrease in EEG amplitude. The presence of spindle activity during periods of low temperature and metabolic rate is unexplained. It is important to note that this spindle like activity is at significantly lower frequency than sleep spindles that normally occur early non-REM sleep (stage 2) which are generated by neurons in the reticular region of the thalamus. [36] The function and physiology of these lower frequency oscillations occurring during hibernation at relatively lower temperatures remains unknown and further work will be needed to establish what role such activity may play in the process of hibernation. [18]–[19] We further note that, if it is confirmed that these periods of ultra-low voltage EEG activity are a universal marker of decreased brain metabolic activity during hibernation, they would be of high interest as the target state for attempts to induce hibernation-like states in animals and ultimately humans, an effort motivated by the multitude of medical and societal benefits which could be achieved if it were possible to safely and reversibly induce such a state [37]–[40].

Episodes of REM were abundant in all of the animals studied except the one studied in the DLC where few epochs including REMs were seen. We hypothesize that this reflects that the animal was exposed to ambient temperature that was constant and below the level where REMS occurred in many of the other animals studied (See Figure 2 and Table 1). These periods of REM sleep-like activity occurring in a state other than sleep in euthermic animals have never been previously described. Evidence that these episodes reflected REM sleep is that they all met the criteria of: 1) quiescence; 2) including at least 1 rapid lateral eye movement; and 3) having diminished muscle activity compared with other states (beta and gamma power were significantly lower than during other epochs of data). In mammals, REM sleep propensity is generally greatest during the last half of the usual sleep period. [41] That these REM-like periods were seen both during the usual wake and sleep periods of the circadian cycle for these animals supports that this is not typical REM sleep but is a phenomenon unique to hibernation. The novelty of our observation that REM-like activity occurs during hibernation in C. medius suggests the need to follow-up on this work and confirm that this is indeed REM sleep by determining if other features typical of REM sleep are present, including sexual arousal and autonomic fluctuations [42].

There are few states marked by the relative preponderance of REM compared with non-REM sleep seen in these torporing primates including sleep following REM deprivation, severe hypothyroidism, and the effects of a few medications such as cholinergic agonists. [43]–[44] The link with hypothyroidism is of particular interest considering that some hibernators are believed to decrease thyroid hormone production prior to and during hibernation, raising the possibility that some of the differences in findings related to hibernation in different species might reflect differences in thyroid levels during hibernation. [43] In this regard, it is possible that due to the low thyroid state, non-REM sleep was present during hibernation but we were unable to identify it due to the relative decrease in slow-wave amplitude that occurs when thyroid levels decrease [43].

In terms of the functions and moderators of sleep, our findings confirm a link of sleep with metabolic rate and temperature. Like the evidence that ground squirrels do not manifest classic signs of sleep deprivation when they emerge from hibernation bouts, our findings contradict the long-held view that the drive for non-REM sleep invariably builds up in a homeostatic fashion. [24]–[25], [45]–[49] Non-REM sleep did not occur over at least 5 days without apparent detriment, even when body temperatures were in the normal range for non-hibernation season. This provides strong evidence that the need for sleep as manifested in the homeostatic drive for non-REM is dependent on metabolic rate and body temperature and, when these are decreased from their usual levels, homeostatic sleep drive decreases. This view is consistent with a series of recent studies suggesting that NREM sleep is driven by metabolic activity. [10]–[13] Further studies will be needed to determine whether a rebound of non-REM sleep occurs during periodic arousals from hibernation in dwarf lemurs. However, we saw no evidence for this in the animals studied shortly after being aroused from hibernation for our recordings. Further work will also be needed to determine whether a rebound of NREM sleep might occur over longer periods of observation.

It will also be of interest to carry out additional work correcting for the effects of temperature on the EEG in C. medius. Although prior work suggests that there are relationships between EEG frequency content and temperature, including during torpor, we did not attempt to correct for temperature effects on the EEG [50]–[51] because we believe that when obtaining and scoring the first EEG recordings in an animal it is more conservative to score the data as it is obtained without making any assumptions about how temperature might affect EEG activity based on data collected in other animals.

The most important conclusion is that our findings may indicate differences between primate and non-primate hibernators the importance of which motivate that future studies be carried out which include the recording of body temperature and metabolic rate in more dwarf lemurs in order to confirm our findings.
